# Serum proteomics reveals survival-associated biomarkers in pancreatic cancer patients treated with chemoimmunotherapy

**DOI:** 10.1016/j.isci.2025.112230

**Published:** 2025-03-16

**Authors:** Marco Tognetti, Lopamudra Chatterjee, Nigel Beaton, Kamil Sklodowski, Roland Bruderer, Lukas Reiter, Christoph B. Messner

**Affiliations:** 1 Biognosys, Schlieren, 8952 Zurich, Switzerland; 2 Precision Proteomics Center, Swiss Institute of Allergy and Asthma Research (SIAF), University of Zurich, 7265 Davos, Switzerland; 3 The LOOP Zurich, 8044 Zurich, Switzerland; 4 Swiss Institute of Bioinformatics (SIB), 1015 Lausanne, Switzerland

**Keywords:** Treatment, Body substance sample, Cancer, Proteomics

## Abstract

Immunotherapy has transformed the landscape of cancer treatment but remains largely ineffective for patients with pancreatic ductal adenocarcinoma (PDAC). Some patients, however, show improved outcomes when treated with a combination of immunotherapy and chemotherapy. Here, we conducted deep serum proteome analysis to investigate the protein profiles of PDAC patients and changes during this combinatorial treatment. Utilizing an advanced serum workflow, we quantified 1,011 proteins across 211 samples from 62 patients. Glycolytic enzymes were associated with survival in anti-PD-1-treated patients, with their abundances significantly correlating with expression levels in tumor biopsies. Notably, a set of protein biomarkers was found to be highly predictive of survival in anti-PD-1-treated patients (area under the curve [AUC] = 0.91). Overall, our data demonstrate the potential of deep serum proteomics for precision medicine, offering a powerful tool to guide patient selection for treatment through minimally invasive serum protein biomarker measurements.

## Highlights

• Serum proteomics reveals molecular subgroups of PDAC patients with prognostic value
• Glycolysis is associated with survival in nivolumab-treated patients
• Protein biomarkers predict one-year survival in patients receiving nivolumab treatment

## Graphical abstract

**Figure:**
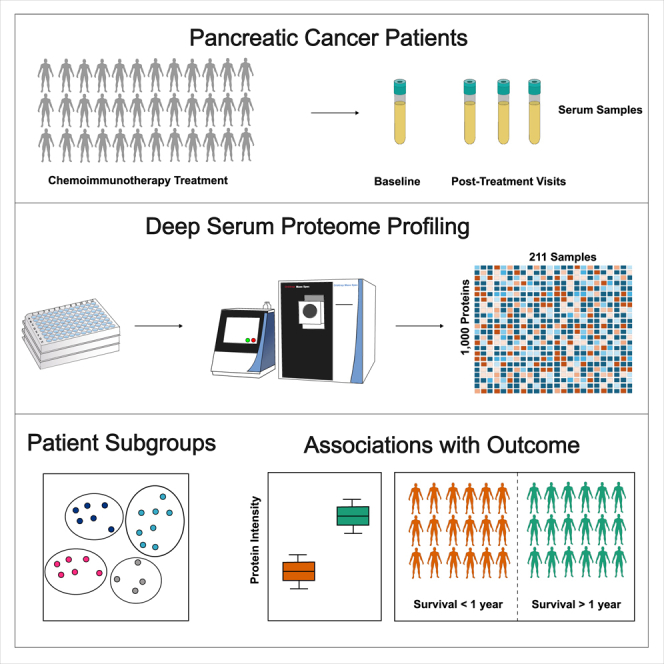

## Introduction

Pancreatic ductal adenocarcinoma (PDAC) is one of the deadliest cancers, characterized by a complex, immunosuppressive tumor microenvironment. Patients with PDAC have short survival times and limited treatment options. While PD-1 inhibitors and other immunotherapies have revolutionized the treatment of many cancer types, PDAC patients generally show unresponsiveness to this type of therapy.^1^ However, a recent study indicates moderate survival improvements with a combination of chemotherapy and immunotherapy in late-stage PDAC.^2^ Notably, a subset of patients, characterized by specific immune cell profiles, has shown survival benefits, particularly with nivolumab (anti-PD-1) treatment.^2^

There is an urgent need to better understand resistance to immunotherapy in PDAC and to identify reliable biomarkers for therapy response. Blood-derived protein biomarkers hold promise for patient stratification due to the ease of blood collection and the cost-effectiveness of protein quantification methods like immunoassays^3^ or targeted mass spectrometry (MS).^4^ However, the discovery of protein biomarkers has lagged behind expectations due to limited sensitivity, throughput, and depth of proteomics technologies. In particular, analyzing low-abundance proteins in plasma or serum is challenging.^5^^,^^6^ The broad dynamic range of neat plasma/serum and limitations in large-scale depletion have constrained the broad application of MS-based plasma/serum proteomics in clinical trials. However, recent developments have enhanced the depth, throughput, and reproducibility of MS-based plasma and serum proteomics.^7^^,^^8^^,^^9^^,^^10^^,^^11^

In this study, we utilized an advanced serum proteomics workflow to analyze samples from a longitudinal clinical trial cohort of metastatic PDAC patients undergoing combined immunotherapy and chemotherapy. This method enabled us to robustly quantify 1,011 proteins across 211 samples. Our findings demonstrate that serum proteomics can detect subtypes and identify prognostic markers for patients receiving anti-PD-1 treatment. Our results highlight the potential of serum and plasma proteomics in supporting clinical trials and advancing precision medicine.

## Results

### Sample cohort and study design

We conducted deep proteome analysis of samples derived from a multi-center phase 2 clinical trial for stage IV PDAC patients as detailed in the work of Padron et al.^2^ We analyzed two treatment arms, namely nivolumab (PD-1 inhibitor)/chemotherapy as well as sotigalimab (CD40 agonistic antibody)/chemotherapy. Samples were longitudinally collected (Figure S1A), with pretreatment sample collection as well as up to four subsequent collections. The number of patient samples analyzed was 30 (nivolumab arm) and 32 (sotigalimab) with patient characteristics listed in Table 1. One-year overall survival was 57.7% and 47.1% for the nivolumab and sotigalimab treatment arm, respectively.

**Table 1:** Patient characteristics

Characteristic	Nivolumab/chemotherapy *n* (%)	Sotigalimab/chemotherapy *n* (%)
Sex: F	12 (40)	11 (34)
Sex: M	18 (60)	21 (66)
Race: White	26 (87)	27 (84)
Race: Asian	3 (10)	4 (13)
Race: Black or African American	0 (0)	1 (3)
Race: other	1 (3)	0 (0)
Age: max	75 (NA)	78 (NA)
Age: median	63.5 (NA)	61.5 (NA)
Age: min	47 (57NA)	35 (NA)

Data are shown as numbers and percentages.

### Proteomics technology and quality control

**Figure 1:**
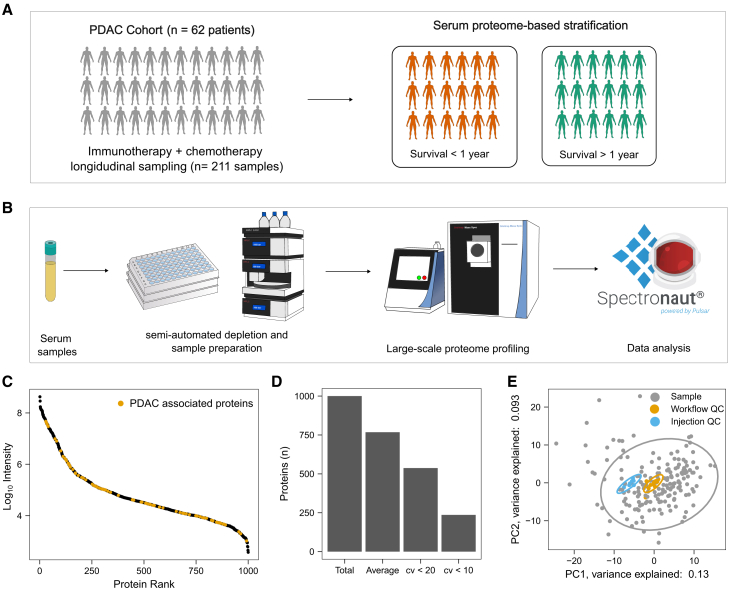
Study design, methodology, and quality control (A) Study design: Multi-center clinical trial in patients with late-stage metastatic PDAC. Patients received immunotherapy (anti-PD-1 or CD40 agonistic antibody) combined with chemotherapy (gemcitabine/nab-paclitaxel). This study aimed to find pretreatment protein markers that can classify the patients into survival groups. (B) Large-scale deep serum proteomic profiling. Samples were processed in 96-well plate format. High-abundant proteins were depleted with a semi-automated workflow, and samples were processed with an adapted and semi-automatized protein aggregation capture (PAC) protocol. Raw data were processed with Spectronaut 16 (Biognosys). (C) Proteins are identified across 4 orders of magnitude. Identified proteins are plotted by decreasing abundance rank (x axis). Proteins that were previously associated with PDAC are labeled.^9^ Protein intensities were averaged across the whole process QC samples, and the y axis is log_10_ transformed. (D) Number of identifications and quantification. The number of identified proteins across all measured samples (total) and the average number of identified proteins as well as proteins quantified below 20% and 10% coefficient of variations are shown. Coefficients of variation were calculated for whole process QC samples. (E) The main variation in the dataset is biological and not technical. Principal-component analysis shows that the spread in the first 2 components is mainly driven by the patient samples while the QC samples are close to each other.

We analyzed a total of 211 samples and aimed to maximize the depth of the serum proteome analysis to ensure the identification of any potential predictive biomarkers associated with PDAC and immunotherapy. PDAC plasma biomarkers have been previously shown to be low abundant.^9^ We thus employed a workflow capable of deep proteome profiling of large cohorts using a semi-automated depletion and an optimized data-independent acquisition (DIA) scheme (Figure 1B). To control for instrument and sample preparation variability, we introduced 9 injection quality control (QC) samples (repeat injections of the same sample) and 13 whole process QC samples (independently prepared samples). After stringent filtering, we identified 1,001 proteins in the QC samples, ranging across more than four orders of magnitude (Figure 1C). The average number of proteins identified per sample was 767 (Figure 1D). 214 out of the identified proteins were enriched in the liver; 21 were enriched in the pancreas; 277 were annotated as secreted; and 651 had annotations other than pancreas, liver, or secreted (Figure S1D).

Precise quantification was achieved, with 537 and 236 proteins quantified below coefficients of variation (CVs) of 20 and 10, respectively (Figure 1D). The median CV values for all identified proteins were 11.3%, 17.8%, and 42.7% for instrument QCs, whole process QCs, and patient samples, respectively (Figure S1B). These results indicate that technical reproducibility is much lower compared to the variability observed in the biological samples. This is further illustrated in the first two principal components of a principal-component analysis (PCA) analysis, where the control samples are in close distance, while the patient samples are more spread out (Figure 1E). Further, no bias or batch effect according to the site of collection was visually observed in a PCA analysis (Figure S1C). Overall, the applied technology has, to the best of our knowledge, facilitated the most comprehensive and in-depth analysis of PDAC serum samples using proteomics to date.

### Unsupervised analysis reveals PDAC subtypes with prognostic value

**Figure 2:**
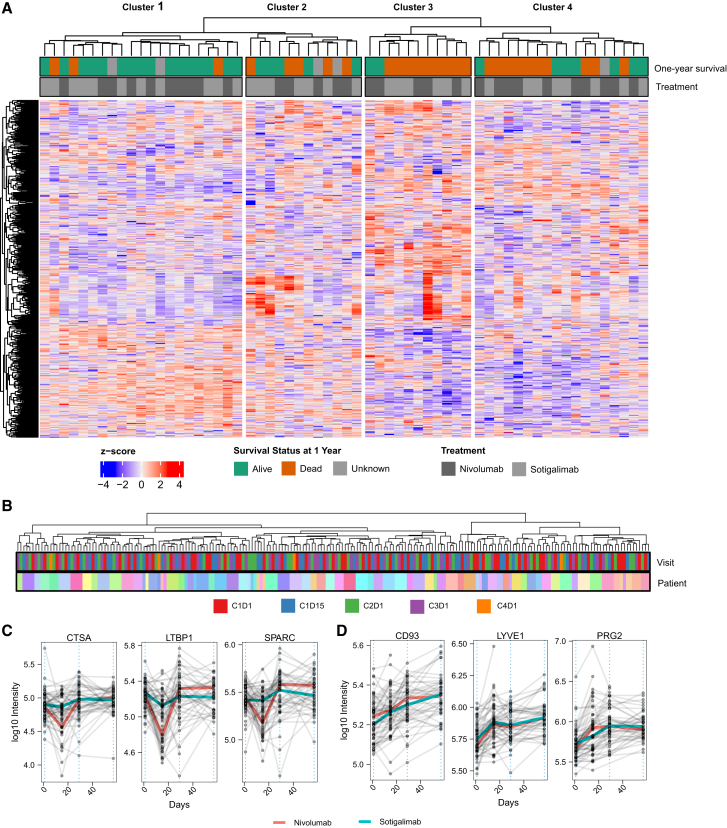
Proteomic analysis reveals patient subgroups and treatment-specific protein changes (A) Unsupervised analysis of pretreatment serum samples identifies patient clusters related to prognosis. The heatmap is based on *Z* scores of 566 protein quantities (proteins identified in at least 80% of patients). Patients were split into four groups based on hierarchical clustering. The top panel shows one-year survival and received treatment. The one-year survival of patients that were lost to follow-up within the first year is indicated as unknown. (B) The clustering of samples is mainly driven by the inter-variability across patients rather than the time of sample collection. Unsupervised clustering was performed using the same method as described in (A) but with all time points included. See Figure S1A for sampling scheme and time points. (C) Protein changes specific to nivolumab treatment. The three most significantly differentially expressed proteins at day 15 between patients receiving nivolumab and those receiving sotigalimab. The dashed line indicates the beginning of each treatment cycle (see Figure S1A for the sampling scheme). Each point represents a patient at a specific time point, and samples from the same patient are connected with a gray line. The colored lines connect the median values from each time point for nivolumab (red)- and sotigalimab (light blue)-treated patients. (D) Longitudinal protein changes across treatment cycles. The top three differentially expressed proteins between the first cycle (pretreatment, C1D1, day 1) and the third cycle (C3D1, day 57) are shown. Each point represents a patient at a specific time point, and samples from the same patient are connected with a gray line. The colored lines connect the median values from each time point for nivolumab (red)- and sotigalimab (light blue)-treated patients.

**Figure 3:**
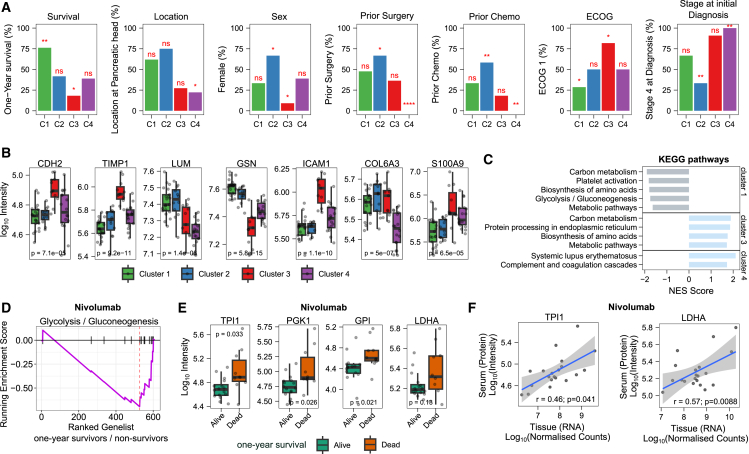
Clinical and molecular differences driving subgroup classification and prognosis (A) Associations of metadata with the observed clusters. Metadata include one-year survival, cancer location (head or body/tail), sex (female/male), prior surgery, prior chemotherapy, ECOG status (1/0), and stage at initial diagnosis. *p* values were calculated using Fisher’s exact test. Asterisks indicate significance levels (∗∗∗∗ for *p* value ≤ 0.0001; ∗∗∗ for *p* value ≤ 0.001; ∗∗ for *p* value ≤ 0.01; ∗ for *p* value ≤ 0.05). Tests were performed as binary (e.g., significant enrichment of patients with stage 4). Numbers of patients in each cluster are shown as percentages (*n* = 62). Data are shown for both treatment arms. See Figure S3A for the non-binary data. (B) Proteins differentially expressed across the clusters. The y axis is log_10_ transformed. *p* values show ANOVA results (not adjusted). Boxplots display the first and third quartiles, as well as the median (thick line); whiskers extend to the most extreme data point no more than 1.5× the interquartile range from the box. (C) Clusters are enriched in metabolic pathway changes. Gene set enrichment analysis (GSEA) was performed for each of the clusters using KEGG pathways.^13^^,^^14^ The ranked gene lists were generated by comparing the protein intensities of the samples from the respective cluster to the protein intensities of all other samples (fold changes). Terms enriched with an adjusted *p* value below 0.05 are shown. (D) Pretreatment levels of *Glycolysis/Gluconeogenesis* are related to survival. Comparison of pretreatment protein levels between patients who survived one year and those who did not, within the nivolumab treatment group. The plot displays the running enrichment score (violet line) for *Glycolysis/Gluconeogenesis*-associated proteins, based on a ranked list of genes generated from fold changes between patients who survived one year and those who did not. (E) Pretreatment glycolytic protein levels are related to survival in patients treated with nivolumab. Boxplots of selected proteins associated with *Glycolysis/Gluconeogenesis* for patients treated with nivolumab. The y axis is log_10_ transformed. Boxplots show the first and third quartiles, as well as the median (thick line); whiskers extend to the most extreme data point no more than 1.5× the interquartile range from the box. *p* values were calculated with a t test (not adjusted). The glycolytic proteins showed no significant association with prior surgery (Figure S3I). (F) Correlation of protein levels (serum) and RNA expression (tissue biopsies). Pearson correlation coefficient (r) and respective *p* value are shown. RNA data were reanalyzed from Padrón et al.^2^ Shaded area represents 95% confidence interval.

First, we investigated whether we can categorize PDAC patients into subgroups based on their proteome profiles. We performed unsupervised hierarchical clustering and divided the patients into 4 groups (Figure 2A). Indeed, we identified subgroups that significantly differed in survival. For example, 16 out of 19 patients, for whom one-year survival data were available, survived longer than one year in Cluster 1. Thus, patients with a serum profile related to Cluster 1 have a significantly better prognosis (Figure 3A). Considering only patients receiving nivolumab, the one-year survival was even 100% in this cluster. Cluster 3, on the other hand, is characterized by a particularly low survival rate, with only 2 out of 11 surviving for 1 year. Cluster 3 is also enriched with male patients (Figure 3A) and elevated levels of TIMP1 (Figure 3B). Interestingly, it has been documented that TIMP1 levels, gender, and clinical outcomes are interrelated.^12^

We also identified associations with Eastern Cooperative Oncology Group (ECOG) performance status at screening, where all patients had either ECOG status 0 or 1. Cluster 3, associated with poorer outcomes, showed a higher prevalence of patients with an ECOG status of 1. In contrast, Cluster 1, which is linked to better prognosis, had a lower proportion of patients with ECOG status 1 (Figure 3A).

While all patients were enrolled at stage IV, some were initially diagnosed at stages I–III. We found that Cluster 2 had a significantly higher proportion of patients initially diagnosed at stages I–III, whereas Cluster 4 was enriched with patients who had stage IV at initial diagnosis (Figures 3A and S3A). However, no significant association between the stage at diagnosis and survival was found (Fisher’s exact test, *p* > 0.05).

Furthermore, patients with surgery were significantly enriched in Cluster 2, where 8 out of 12 patients had undergone surgery, compared to Cluster 4, where none out of 18 patients had surgery (Figure 3A). Overall, patients with prior surgery had a better prognosis (Fisher’s exact test, *p* value = 0.025; Figure S3H). Among the top changed proteins in patients that had surgery/prior treatment are Protein S (PROS1) and C-type mannose receptor 2 (MRC2), both of which are linked to fibroblast-extracellular matrix (ECM) organization or connective tissue cells’ ECM organization, indicating activated fibroblasts and other connective tissue cells involved in ongoing wound healing and tissue repair. Additionally, we observed the downregulation of C4b-binding protein beta chain (C4BPB) (Figure S3B).

Further, we found that the clusters partially reflect cancer location, as previously subtyped into head and body/tail^15^^,^^16^; Cluster 4 was significantly enriched with patients whose cancer was located in the body/tail.

To compare the observed clusters with the previously established basal-like and classical subtypes, we calculated the Purity Independent Subtyping of Tumors (PurIST) classifier^17^ using transcriptomics data from biopsies available for a subset of patients (from Padrón et al.^2^). We found that the classical subtype was associated with longer overall survival (Figure S2B), consistent with previous findings.^17^ Cluster 3 had the highest proportion of basal-like subtype patients; however, this was not statistically significant (*p* = 0.08; Fisher’s exact test) (Figure S2C).

### Longitudinal serum proteome changes

We then investigated the protein changes over time and during the course of treatment (see Figure S1A for the sampling scheme). Overall, the longitudinal changes were small compared to the inter-individual variability, with samples mainly clustering by patient and not by collection time (Figure 2B). However, specific proteins showed longitudinal changes, with 31 proteins identified as having significant changes during the first treatment cycle (day 15 compared to day 1; adjusted *p* value < 0.01; see STAR Methods; Table S1). Consistent with previous omics data,^2^ we found that the nivolumab treatment showed stronger responses than the sotigalimab treatment (Figure S2A). Four proteins were found to be significantly different between the nivolumab- and sotigalimab-treated patients (adjusted *p* value < 0.01), such as Cathepsin A (CTSA), Latent transforming growth factor beta-binding protein 1 (LTBP1), and Secreted protein acidic and cysteine rich (SPARC) (Figure 2C; Table S2).

We further analyzed the proteome change after two treatment cycles by comparing it to the baseline (C1D1 vs. C3D1). We identified 40 proteins that significantly changed between baseline and prior to the third treatment cycle (Table S3, adjusted *p* value < 0.01; see STAR Methods). Among these, the most significantly changing proteins included Complement component C1q receptor (CD93), Bone marrow proteoglycan (PRG2), and Lymphatic vessel endothelial hyaluronan receptor 1 (LYVE1).

### Subgroups are driven by known markers of PDAC pathogenesis

Next, we analyzed the molecular differences between the identified subgroups (as defined in Figure 2A). Through gene set enrichment analysis comparing the different clusters, we identified several significantly enriched terms, including *cadherin binding*, *RNA binding*, or cell *adhesion molecule binding* (Figure S3C). Notably, cadherin binding and N-cadherin (CDH2) are among the most altered terms and proteins across the clusters (Figures 3B and S3C). N-cadherin is linked to the epithelial-mesenchymal transition (EMT), specifically by switching from E-cadherin to N-cadherin.^18^^,^^19^ EMT has been associated with metastatic phenotypes, progression, and drug resistance in various cancers, including pancreatic carcinomas.^20^^,^^21^^,^^22^^,^^23^^,^^24^

Further, we found many of the most significantly changed proteins between the clusters that have been previously associated with PDAC based on proteomics measurements (Table S4). For example, Gelsolin (GSN); Lumican (LUM); Metalloproteinase inhibitor 1 (TIMP1)^25^^,^^26^; Intercellular adhesion molecule 1 (ICAM1)^27^; Collagen, type VI, alpha 3 (COL6A3); or Protein S100-A9 (S100-A9).^28^ Notably, LUM and GSN were previously found to distinguish proliferative and inflammatory subtypes in proteomics analyses of liver metastases.^29^

Additionally, an enrichment analysis on Kyoto Encyclopedia of Genes and Genomes (KEGG) pathways revealed changes in carbon metabolism, particularly in *glycolysis/gluconeogenesis*, across the clusters (Figure 3C). For instance, we observed that Fructose-bisphosphate aldolase (ALDOA), Glyceraldehyde-3-phosphate dehydrogenase (GAPDH), or Glucose-6-phosphate isomerase (GPI) have lower levels in Cluster 1 compared to the other clusters (Figure S3D).

### Glycolysis is related to survival in nivolumab-treated patients

As patients in Cluster 1 exhibited low levels of glycolytic enzymes and demonstrated favorable prognosis with nivolumab treatment, we next analyzed the relationship between glycolysis, treatment, and prognosis. Indeed, we found a significant association between survival and *Glycolysis/Gluconeogenesis* in patients receiving nivolumab (Figures 3C and 3D). For example, nivolumab-treated patients with lower levels of GPI, Triosephosphate isomerase (TPI1), Phosphoglycerate kinase 1 (PGK1), or L-lactate dehydrogenase A chain (LDHA) exhibited better survival (Figure 3E). These findings suggest increased glycolysis and lactate production in patients with poorer outcomes under nivolumab treatment. The relevance of lactate in cancer, as well as the function of lactate dehydrogenase (LDH) in T cells and the immune system, has been previously described.^30^^,^^31^ Indeed, high serum LDH levels have been identified as a biomarker for poor outcomes in anti-PD-1-treated melanoma patients.^32^^,^^33^^,^^34^

Interestingly, we observed one glycolytic enzyme changed in the opposite direction to the other glycolytic enzymes: ALDOB was increased rather than decreased in the survival group (see Figures 3C and S3F). This contrast with isozyme A suggests that ALDOA/ALDOB ratio could be a promising biomarker for survival (Figure S3F).

We also observed that certain glycolytic enzymes, such as TPI1 or PGK1, were particularly elevated in a subset of former smokers with poor outcomes (*p* value < 0.01, ANOVA) (Figure S3G). Interestingly, an association of smoking history with a distinct metabolic subtype has been previously reported by a proteomics analysis of liver metastases.^29^

To test whether the changes in metabolic enzymes in the serum originate from metastatic tissue, we reanalyzed the previously published transcriptomics dataset. Indeed, we found that the expression of the metabolic enzymes LDHA, GPI, PGK1, and TPI1 significantly correlates with their serum protein levels (Figures 3F and S3E), suggesting that the metabolic state within metastatic sites can be detected through serum proteomics.

### One-year survival prediction in nivolumab-treated patients

**Figure 4:**
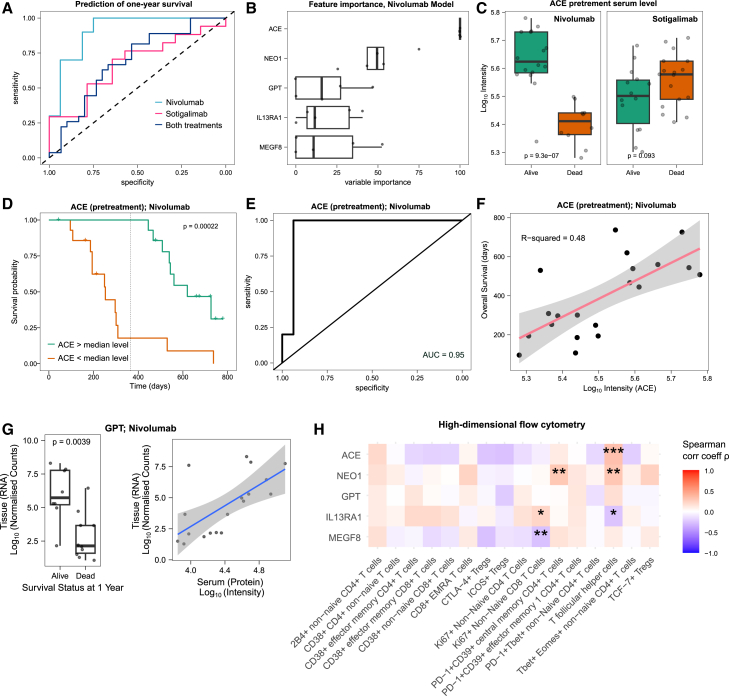
Survival prediction from pretreatment serum proteome (A) Prediction of one-year survival for patients receiving nivolumab, sotigalimab, or either of both. Generalized linear models with elastic net regularization were applied. To avoid data leakage between the test and train datasets, we employed nested 5-fold cross-validation and generated five models, each applied to a distinct hold-out dataset (each comprising 20% of the entire dataset). (B) Feature/variable importance. The five most important proteins in the regression model for predicting survival in nivolumab-treated patients. Feature importance was calculated for all of the five train sets in the nested design. Proteins are ranked by median importance (from top to bottom) and scaled to have a maximum value of 100. The proteins were found to be not significantly associated with prior cancer surgery (Figure S4). Boxplots display the first and third quartiles, as well as the median (thick line); whiskers extend to the most extreme data point no more than 1.5× the interquartile range from the box. (C) High pretreatment ACE protein levels are associated with longer survival in patients receiving nivolumab (left panel) but not in patients receiving sotigalimab (right panel). The y axis is log_10_ transformed. Boxplots display the first and third quartiles, as well as the median (thick line); whiskers extend to the most extreme data point no more than 1.5× the interquartile range from the box. *p* values were calculated with a t test (not adjusted). (D) Patients with high pretreatment ACE levels have significantly longer survival. The Kaplan-Meier survival analysis compares patients with high pretreatment levels of ACE (above median) to those with low pretreatment levels of ACE (below median). The *p* value was calculated with a log rank test. (E) One-year survival classification based on pretreatment ACE levels. Receiver operating characteristic (ROC) curve based on pretreatment ACE protein levels for classifying patients with survival time above and below one year. Patients who were lost to follow-up within the first year were not considered. (F) ACE levels correlate with overall survival (OS). Correlation of ACE protein intensities with overall survival in patients treated with nivolumab. Only patients that have been reported as having died were considered. The x axis is log_10_ transformed. (G) GPT correlates significantly between serum and tissue biopsy samples. Tissue RNA expression levels in biopsies are shown for different survival statuses, along with their correlation to the respective protein levels in serum for patients receiving nivolumab/chemotherapy. Biopsy data were obtained from the previous study by Padrón et al.^2^ Boxplots (left panel) display the first and third quartiles, as well as the median (thick line); whiskers extend to the most extreme data point no more than 1.5× the interquartile range from the box. Shaded area represents 95% confidence interval (right panel). (H) Correlations of the identified markers with high-dimensional flow cytometry data (X50) (reanalyzed from Padrón et al.^2^). Spearman correlation coefficients are shown as a heatmap, and asterisks indicate the significance level (∗ for *p* ≤ 0.05, ∗∗ for *p* ≤ 0.01, and ∗∗∗ for *p* ≤ 0.001). Cell populations were defined as previously described.^2^

Next, we investigated the predictability of one-year survival based on the pretreatment serum proteomes. We trained elastic net models for patients treated with nivolumab, sotigalimab, or both groups combined. We used 5-fold nested cross-validation, applying the models to independent hold-out datasets. While the predictive power for the sotigalimab and the combined group was moderate (area under the curve [AUC]: 0.67 and 0.69, respectively), we found high predictability of the one-year survival for nivolumab-treated patients (AUC = 0.91) (Figure 4A).

To understand what drives these survival predictions for nivolumab-treated patients, we performed a feature importance analysis for each of the 5 nested models (Figure 4B). We found that Angiotensin I converting enzyme (ACE), Neogenin (NEO1), and Alanine aminotransferase 1 (GPT) were the most important features for the elastic net models. Interestingly, previous studies have reported high GPT levels as markers for outcomes in patients receiving anti-PD-1 treatment,^35^ and the receptor NEO1 is indirectly connected to PD-1 via binding the repulsive guidance molecule b (RGMb), which binds to the PD-1 ligand PD-L2.^36^^,^^37^

The most important protein in all the 5 models was ACE. A statistical analysis, adjusting for age and sex, revealed that ACE levels were indeed significantly different in patients with different survival statuses (adjusted *p* value: 0.016, Table S5; Figure 4C). Indeed, patients with high ACE levels were found to live significantly longer (Figures 4D and 4F), and ACE intensities discriminate patients based on one-year survival time with an AUC of 0.95 (Figure 4E). Furthermore, a comparison with sotigalimab showed that these differences are specific to anti-PD-1, as they were not observed and even changed in the opposite direction, for patients treated with CD40 agonist antibodies (Figure 4C).

To investigate whether the source of the observed changes in these proteins is the metastatic tissue, we analyzed the correlation with transcriptome data (from Padrón et al.^2^). While no significant correlation was observed between tissue expression and the proteins ACE, NEO1, MEGF8, or IL13RA1 (*p* value > 0.05), a significant correlation was identified between RNA levels (from tissue biopsies) and protein levels (from serum) for GPT (*p* value = 0.039; Pearson correlation r = 0.64; Figure 4G).

We further explored possible associations of these proteins with immune cells by performing a correlation analysis using data from high-dimensional flow cytometry (X50) (from Padrón et al.^2^) across all time points and samples (Figure 4H). Several significant correlations emerged. For instance, ACE, NEO1, and IL13RA1 showed either positive or negative correlations with T follicular helper cells, which had the strongest predictive value for survival in the nivolumab/chemotherapy treatment arm of the previous study.^2^ NEO1 also correlated with PD-1^+^CD39^+^ central memory CD4^+^ T cells, while IL13RA1 and MEGF8 correlated with Ki67+ non-naive CD8 T cells.

## Discussion

MS-based plasma and serum proteomics have made considerable progress in recent years, with increased throughput and robustness.^5^^,^^7^^,^^8^ However, its application in analyzing samples from clinical trials, particularly for studying treatment responses, has remained limited, despite its potential to drive more personalized treatments. In this study, we applied an advanced MS-based workflow for deep and large-scale proteomics^9^ to profile 211 serum samples from a clinical trial for PDAC patients receiving immunotherapy combined with chemotherapy.^2^ This work highlights the potential of incorporating such advanced proteomics technologies into clinical trials and adds an additional layer to previously collected multi-omics data for this particular clinical trial.^2^

We demonstrated that unsupervised analysis of serum proteomes can identify subgroups of PDAC patients with different survival. Multiple molecular subtyping schemes have been previously proposed for PDAC employing genomics, transcriptomics, or proteomics approaches on tissue samples.^29^^,^^38^^,^^39^^,^^40^^,^^41^ Notably, the differentiation into basal-like and classical subtype has been shown to be associated with survival across several studies.^17^ Reanalyzing existing transcriptomics data, we observed that this classification revealed distinct survival groups (Figure S2B). However, the overlap with serum proteomics clusters was not statistically significant (Figure S2C), and a larger sample size would be needed to investigate this overlap further.

Our study showed that pretreatment levels of glycolytic enzymes in serum are associated with survival in patients treated with nivolumab. We demonstrated that the abundance of these enzymes in serum is significantly correlated with their expression levels in tumor biopsies. The relationship between tumor cell glycolysis, the tumor microenvironment, and immune suppression has been studied and documented,^42^^,^^43^^,^^44^ including PDAC.^40^ Indeed, previous studies have linked increased glycolysis in tumors to poorer prognosis in melanoma patients treated with anti-PD-1.^45^ Our findings build upon these observations by demonstrating that differences in metabolism, indicated by different enzyme concentrations, are detectable not only within tumor cells and their microenvironment but also in serum.

We further demonstrated that our data aid in predicting survival in hold-out datasets. While the applied machine learning model performed well for nivolumab-treated patients, it performed only moderately for patients treated with sotigalimab, suggesting that nivolumab may be overall more effective.

We found that ACE is a particularly promising biomarker for predicting survival in anti-PD-1-treated patients. ACE is mainly known for its role in blood pressure regulation.^46^ However, functions of ACE and the renin-angiotensin system have also been linked to cancer, tumor microenvironment, and (cancer) immunity.^47^^,^^48^^,^^49^ Additionally, Angiotensin II, which is converted by ACE from Angiotensin I,^50^ has been reported to up-regulate the Vascular endothelial growth factor (VEGF) expression,^51^ which has been linked to resistance to anti-PD-1/PD-L1 treatments.^52^^,^^53^ The impact of ACE inhibitors (ACEi) and angiotensin receptor blockers (ARBs) on cancer risk^48^^,^^54^^,^^55^ and treatment outcomes^56^^,^^57^ has been a subject of debate. For example, Medjebar et al. reported impaired outcomes for non-small cell lung cancer (NSCLC) patients when anti-PD-1 treatment is combined with ACEi,^57^ while Suh et al. found improved outcomes when combining anti-PD-1 with renin-angiotensin system (RAS) blockers for patients with melanoma and NSCLC.^56^ Thus, while our study highlights ACE as a potential biomarker for anti-PD-1 patient selection, the functional role of ACE in cancer and cancer immunology warrants further investigation.

Overall, our study serves as a proof of concept for the feasibility of large-scale serum proteomics in clinical trials, highlighting its potential for patient selection and treatment personalization in cancer therapy.

### Limitations of the study

While our findings are promising, the study has several limitations. The absence of a validation cohort limits the generalizability of our results, and, while this is one of the largest PDAC cohorts analyzed using proteomics, the sample size remains relatively small. The identified biomarkers show correlations with survival but not causation, necessitating further mechanistic studies to understand their roles. Additionally, a chemotherapy-only control arm would be necessary to attribute biomarkers to immunotherapy or chemotherapy effects.

## Resource availability

### Lead contact

Further information and requests for resources and reagents should be directed to and will be fulfilled by the lead contact, Christoph B. Messner (christoph.messner@siaf.uzh.ch).

### Materials availability

This study did not generate new materials.

### Data and code availability

• Raw MS data have been deposited to the ProteomeXchange Consortium (http://proteomecentral.proteomexchange.org) via the MassIVE repository. The dataset identifier is listed in the key resources table. The processed dataset derived from the raw data has been deposited at Mendeley Data, and the link is listed in the key resources table. This paper contains analyses that used existing, publicly available data. The identifiers for the datasets are also listed in the key resources table.
• No custom software codes were generated as part of this study. All analyses were conducted in R, using standard, publicly accessible packages obtained either through GitHub (https://github.com/) and the Comprehensive R Archive Network (CRAN, https://cran.r-project.org/) or via Bioconductor (https://www.bioconductor.org/).
• Any additional information required to reanalyze the data reported in this paper is available from the lead contact upon request.

## Acknowledgments

We thank Mirjam Schenk for her help and discussion. We thank the Parker Institute for Cancer Immunotherapy for providing the serum samples, especially Deena M. Maurer, Lacey J. Padrón, Jia Xin Yu, Theresa M. LaVallee, and Diane Da Silva.

The work was partly supported by the Swiss canton of Grisons, The LOOP Zurich, and the 10.13039/501100008949Uniscientia Stiftung.

## Author contributions

M.T. carried out the experiments. M.T., K.S., R.B., and C.B.M. processed, analyzed, and visualized the data. L.C. and N.B. contributed to interpretation of the results. R.B., L.R., and C.B.M. supervised the study. C.B.M. wrote the paper with contributions from all authors.

## Declaration of interests

The authors R.B., M.T., N.B., and L.R. are full-time employees of Biognosys AG (Schlieren-Zurich, Switzerland). Spectronaut is a trademark of Biognosys AG. C.B.M. is an advisor and shareholder of Eliptica Ltd (London, UK).

## Declaration of generative AI and AI-assisted technologies in the writing process

During the preparation of this work, the authors used ChatGPT 4.0 (OpenAI) to improve the readability and quality of the writing after the initial draft. After using this tool, the authors reviewed and edited the content as needed and take full responsibility for the content of the publication.

## STAR★Methods

### Key resources table

**Table:** 

REAGENT or RESOURCE	SOURCE	IDENTIFIER
**Chemicals, peptides, and recombinant proteins**		

iRT peptides	Biognosys	Cat#Ki-3002-b

**Deposited data**		

Raw proteome data	This study	ProteomeXchange: PXD053231
Processed proteome data	This study	Mendeley Data: https://doi.org/10.17632/h2fr3nwzc6.1
RNAseq data	Padrón et al.^2^	https://github.com/ParkerICI/prince-trial-data
High-dimensional flow cytometry data (X50)	Padrón et al.^2^	https://github.com/ParkerICI/prince-trial-data
Full GO term annotation	Gene Ontology Consortium	http://current.geneontology.org/products/pages/downloads.html
KEGG	Kanehisa and Goto^13^; Kanehisa^14^	https://www.genome.jp/kegg/

**Software and algorithms**		

Proteomics data analysis via Spectronaut (version 16.0.220606, Biognosys)	Bruderer et al.^58^	https://biognosys.com/
R Statistical Computing Software	The R Foundation	https://www.r-project.org/
tidyverse	Wickham et al.^59^	https://cran.r-project.org/web/packages/tidyverse/
treeClust R package	Buttrey and Whitaker^60^	https://CRAN.R-project.org/package=treeClust
caret R package for regression modeling	Kuhn et al.^61^	https://CRAN.R-project.org/package=caret
edgeR R package	Robinson et al.^62^	https://bioconductor.org/packages/edgeR
PurIST classification	Rashid et al.^17^	https://github.com/naimurashid/PurIST
pROC R package	Robin et al.^63^	https://xrobin.github.io/pROC/
ComplexHeatmap R package	Gu et al.^64^	https://bioconductor.org/packages/ComplexHeatmap/
Survival R package	Therneau & Grambsch^65^	https://CRAN.R-project.org/package=survival
survminer R package	Kassambara et al.^66^	https://CRAN.R-project.org/package=survminer
DIA-NN R package	Demichev et al.^67^	https://github.com/vdemichev/diann-rpackage
clusterProfiler	Väremo et al.^68^	https://bioconductor.org/packages/clusterProfiler/
limma R package	Ritchie et al.^69^	https://bioconductor.org/packages/limma/

### Experimental model and study participant details

#### Cohort characteristics

The samples were collected as part of a phase 2 trial as previously reported.^2^ Patient samples from the treatment arm receiving sotigalimab, nivolumab, and chemotherapy were not measured. The trial was randomized, open-label, and multi-center. All patients were enrolled at stage IV. Information on sex, age, race, and cancer location (pancreas body, pancreas head, or pancreas tail) is provided in Table 1. The study was conducted in accordance with the principles of the Declaration of Helsinki and International Conference on Harmonisation Good Clinical Practice guidelines. All patients provided written informed consent before enrollment.^2^ The study protocol, inclusion criteria, and statistical analysis plan have been reported by Padrón et al.^2^

### Method details

#### Proteomic sample preparation

Serum samples were profiled using a high-throughput quantitative proteomics workflow at Biognosys (Schlieren-Zurich, Switzerland). All samples were handled and thawed consistently. During aliquoting, a small portion of each sample was pooled and used as a quality control sample for subsequent library generation and to assess quality and batch effects throughout the sample preparation and acquisition. Three processing batches were block-randomized for treatment and site (samples coming from one patient were kept within the same batch but randomized across it). The automated depletion pipeline, composed of sequential depletion and parallel digestion, was performed as previously reported.^9^ Briefly, depletion was performed using the Agilent Multi Affinity Removal Column Human-14, 4.6 x 50 mm (Agilent Technologies) set up on a Dionex Ultimate 3000 RS pump (Thermo Fisher Scientific) and digestion with protein aggregation capture using a KingFisher Flex (Thermo Fisher Scientific).^70^ Quality control samples were depleted within each processing batch.

#### Liquid chromatography–mass spectrometry

For DIA LC-MS measurements, 1 μg of peptides per sample was injected onto an in-house packed reversed-phase column (PicoFrit emitter with 75 μm inner diameter, 60 cm length and 10 μm tip from New Objective, packed the Reprosil Saphir C18 1.5 μm phase (Dr. Maisch, Ammerbuch, Germany)) on a Thermo Fisher Scientific EASY-nLC™ 1200 nano-liquid chromatography system connected to a Thermo Fisher Scientific Orbitrap Exploris 480 mass spectrometer with a Nanospray Flex™ ion source. The DIA method was adopted from Bruderer et al.^71^ and consisted of one full-range MS1 scan followed by 29 DIA segments.

For DDA LC-MS measurements, 1 μg of peptides per sample was injected onto an in-house packed reversed-phase column (PicoFrit emitter with 75 μm inner diameter, 60 cm length and 10 μm tip from New Objective, packed the Reprosil Saphir C18 1.5 μm phase (Dr. Maisch, Ammerbuch, Germany)) on a Thermo Fisher Scientific EASY-nLC™ 1200 nano-liquid chromatography system connected to a Thermo Fisher Scientific Orbitrap Exploris 480 mass spectrometer with a Nanospray Flex™ ion source. Peptides were separated by a 2 h segmented gradient at a flow rate of 250 nL/min with increasing solvent B (0.1% formic acid, 85% ACN) mixed into solvent A (0.1% formic acid, 1% ACN). A top 15 method was used across a scan range of 350–1650 m/z with a full MS resolution of 60,000 (ACG target of 3 × 106 or 20 ms injection time). Dependent MS2 scans were performed with a resolution of 15,000 (ACG target of 2 × 105 or 25 ms injection time) with an isolation window of 1.6 m/z and a fixed first mass of 120 m/z.

Some measurements were repeated due to column clogging and only the repeat injections were considered in subsequent analysis.

#### DIA library generation

High pH reverse phase (HPRP) fractionation was performed using a Dionex UltiMate 3000 RS pump (Thermo Fisher Scientific) equipped with an Acquity UPLC CSH C18 1.7 μm, 2.1x150 mm column (Waters) at 60 °C with 0.3 ml/min flow rate. Prior to loading onto the column, the pH of 300 μg pooled samples was adjusted to pH 10 by adding ammonium hydroxide. The gradient was 1% to 40% solvent B (acetonitrile) in 30 minutes in solvent A (20 mM ammonium formate in water). Fractions were taken every 30 seconds and sequentially pooled into 20 fractions. The fractions were then dried down, resuspended in 1% acetonitrile with 0.1% formic acid, and Biognosys’s iRT kits were spiked in according to the manufacturer’s instruction. Before the DDA mass spectrometric analyses, peptide concentrations were determined, and the samples were centrifuged.

### Quantification and statistical analysis

All statistical analyses were performed using R.^72^ For basic data manipulation and visualization, the tidyverse R packages were used.^59^

Coefficients of variation (CV) were calculated as follows: empirical standard deviations for each protein or precursor were divided by its empirical mean. They were calculated for proteins or precursors identified in at least two replicate measurements. CV values were reported in percentages.

The Kaplan-Meier survival curves were computed with the survfit function within the survival R package^65^ and plotted using the ggsurvplot function from the survminer R package.^66^

The ROC curve was plotted using the ROC function within the pROC R package.^63^

Conversion between UniProt IDs, gene names, and open reading frames (ORFs) was done with the bitr function within the clusterProfiler package^73^^,^^74^ or using the UniProt database.^75^

PCA plots (Figures 1E and S1C) were performed using the prcomp function,^72^ and a complete data matrix was used (all proteins with missing values were removed).

Unsupervised clustering and heatmap generation (Figures 2A and 2B) were performed using the ComplexHeatmap R package.^64^^,^^76^ An Euclidean distance matrix was used, and the ward.D2 implementation within hclust was applied as the clustering method.^72^^,^^77^ Values were centered and scaled (z-scores) for the heatmap.

For Gene Set Enrichment Analysis (GSEA) of the clusters (Figures 3C and S3C), we used Gene Ontology (molecular functions) and KEGG pathways^13^^,^^14^ and employed the gseGO/gseKEGG function within the clusterProfiler package.^73^^,^^74^ Genes were ranked based on their fold-change values (ratio of mean protein intensities within the respective cluster and all other clusters). Multiple testing corrections were applied using the Benjamini-Hochberg procedure.^78^

To analyze one-year survival, patients were split into two groups with overall survival > one year and < one year. Patients who were lost to follow-up within the first year were not considered.

Transcriptome data and high-dimensional flow cytometry data (X50) were reanalysed from Padrón et al.^2^ (see key resources table). Transcriptome raw read counts were normalized using EdgeR R package^62^ to get weighted trimmed mean of the log expression ratios (trimmed mean of M values (TMM)).

#### Database search for library generation

DIA and DDA mass spectrometric data were analyzed using the software SpectroMine (version 4.0.220606.0, Biognosys) using the default settings, including a 1% false discovery rate control at PSM, peptide, and protein levels, allowing for two missed cleavages and variable modifications (N-term acetylation, asparagine and glutamine deamidation, asparagine ammonia-loss and methionine oxidation). The human UniProt.fasta database (Homo sapiens, 2022-01-07, 20,386 entries) was used. For library generation, the default settings were used except for the use of a top 300 precursors per protein filter and the exclusion of modified peptides containing deamidated asparagines and/or glutamines.

#### Quantitative analysis of data-independent acquisition

Raw mass spectrometric data was analyzed using the software Spectronaut (version 16.0.220606, Biognosys) with the same settings as in Tognetti et al.^9^ These are Spectronaut default settings, but Q-value sparse filtering was enabled with a global imputing strategy, the protein LFQ method was set to QUANT 2.0, minor (peptide) grouping set to “Modified Sequence” and a hybrid library comprising all DIA and DDA runs conducted in this study. The default settings include peptide and protein level false discovery rate control at 1% and cross-run normalization using global normalization on the median.

#### Normalization, batch correction, filtering, and protein quantification

Imputed values were filtered and precursors with quantity of 0 were replaced with NAs. Non-proteotypic peptides were removed. Precursors identified in less than 10% of measurements and with a coefficient of variation above 30% in the whole process QC samples were removed. Signal drifts were corrected by fitting a loess function for each precursor across the measurements (span = 0.7) and subtracting the resulting line. Median quantities of each precursor were added back. Protein quantities were calculated using the maxLFQ algorithm^79^ as implemented for DIA data (DIA-NN R package). Signal intensities were log_10_ transformed for differential expression analysis and for plotting.

#### Differential expression analysis

Differential expression analysis between time points and between different survival statuses was performed using the limma R package.^69^ The linear models were fitted protein-wise using the lmFit function within the limma package. Comparisons of interest were extracted using the makeContrasts function.^69^ The t-statistics were computed using the ebayes function, allowing for an intensity trend in the prior variance. Unless otherwise specified, proteins are considered differentially expressed if their adjusted p-value is below 0.05. Age and gender were incorporated into the models for the comparison of one-year survival at pretreatment sampling (Table S5).

To study the response during treatment, we compared C1D15 and C1D1 (contrast matrix) (Table S1). For examining longitudinal responses across treatment, C3D1 was compared against C1D1 (Table S3; Figure 2D). The time point C4D1 was not considered for the differential expression analysis due to the low sample number (n= 4; see Figure S1A).

To investigate differences in response between treatments, we compared protein levels in Nivolumab-treated patients to those in Sotigalimab-treated patients during the first treatment cycle (C1D15) (Table S2; Figure 2C).

For comparing different clusters observed in Figure 2A, we performed ANOVA analysis (Figure 3B).

Unless otherwise specified, multiple testing corrections were applied using the Benjamini-Hochberg procedure.^78^

#### Prediction of one-year survival

For predicting one-year survival, patients were split into two groups with overall survival longer than one year and shorter than one year. Patients who were lost to follow-up within the first year were not considered. We applied generalized linear models with an elastic net regularization^80^ using the glmnet implementation^81^^,^^82^ within the caret R package.^61^ To prevent data leakage between the training and test sets, we employed a nested cross-validation approach. We generated 5 splits, each with an 80/20 train/test ratio. For each split, we trained a model using 5-fold cross-validation and a tune length of 5 for parameter optimization. The importance of features/variables was estimated using the absolute value of the coefficients from the tuned model, as implemented in the varimp function within the caret R package.^61^
