# Supplementary material for: Serum proteomics reveals survival-associated biomarkers in pancreatic cancer patients treated with chemoimmunotherapy
Source: iScience. 2025 Mar 16;28(4):112230. doi: 10.1016/j.isci.2025.112230 (PMC11999289; doi:10.1016/j.isci.2025.112230)
Supplement: Document S1. Tables S1 and S5 and Figures S1–S4 [file mmc1.pdf]

iScience, Volume *28*

Supplemental information

Serum proteomics reveals survival-associated biomarkers in pancreatic cancer patients treated with chemoimmunotherapy

Marco Tognetti, Lopamudra Chatterjee, Nigel Beaton, Kamil Sklodowski, Roland Bruderer, Lukas Reiter, and Christoph B. Messner

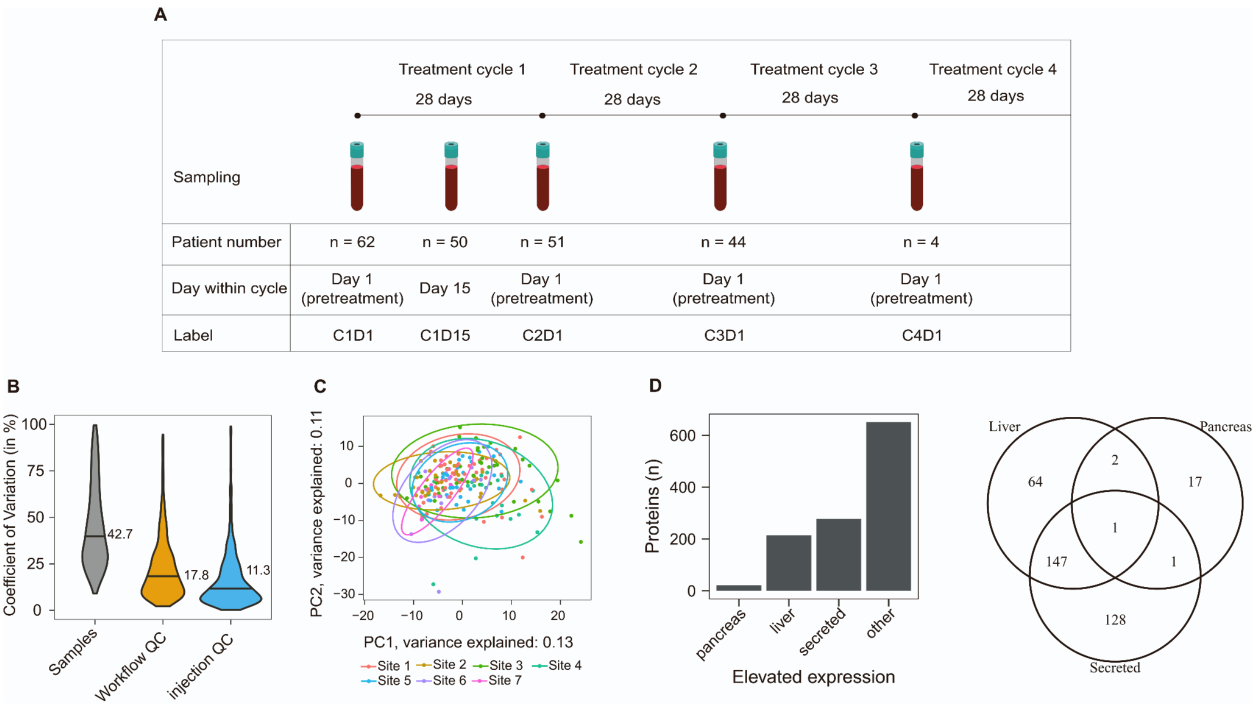

Figure S1, related to Figure 1: A) Detailed overview of sample collection during the multicenter phase 2 clinical trial. **B)** Coefficients of variation (in %) for the samples, workflow QC (pooled before sample preparation), and injection QC (pooled before measurement) across 292 injections. Numbers refer to median values. **C)** PCA plot shows no batch effect resulting from the collection site. Colors differ between collection sites. **D)** Proteins annotated according to the human protein atlas (proteinatlas.org)[1,2]. The criteria used were as follows: tissue enriched (at least four-fold higher mRNA level in the liver/pancreas compared to any other tissues); group enriched (at least four-fold higher average mRNA level in a group of 2-5 tissues compared to any other tissue); and tissue enhanced (at least four-fold higher mRNA level in the liver/pancreas compared to the average level in all other tissues).

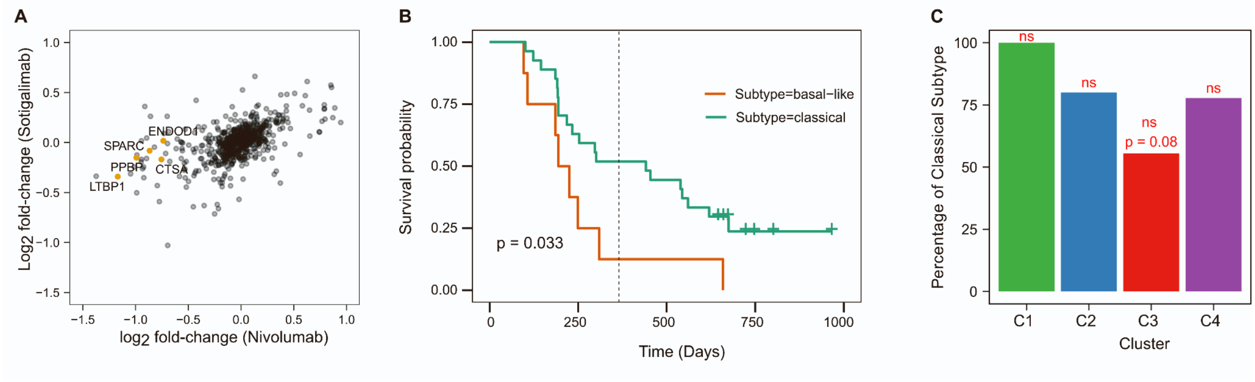

Figure S2, related to Figure 2: A) Nivolumab shows stronger treatment responses compared to Sotigalimab. Protein changes at day 15 (C1D15) are plotted as fold-changes (compared to baseline (C1D1)) and compared between Nivolumab (x-axis) and Sotigalimab (y-axis). Proteins that are significantly different between the treatments

are colored and labeled. **B) The basal-like subtype is associated with shorter overall survival.** Classical and basal-like subtypes were assigned using the Purity Independent Subtyping of Tumors (PurIST) classifier [3]. Among the 35 samples where biopsies were available, we identified 8 basal-like and 27 classical subtypes. This analysis is based on the reanalysis of previously published transcriptome data[4]. **C) Associations of basal-like and classical subtypes with the observed clusters (Figure 2A).** P-values were calculated using Fisher's exact test. Significance levels are indicated (ns for p-value > 0.05). Number of patients in each cluster are shown as percentages (n = 35). This analysis is based on the reanalysis of previously published transcripomics data[4].

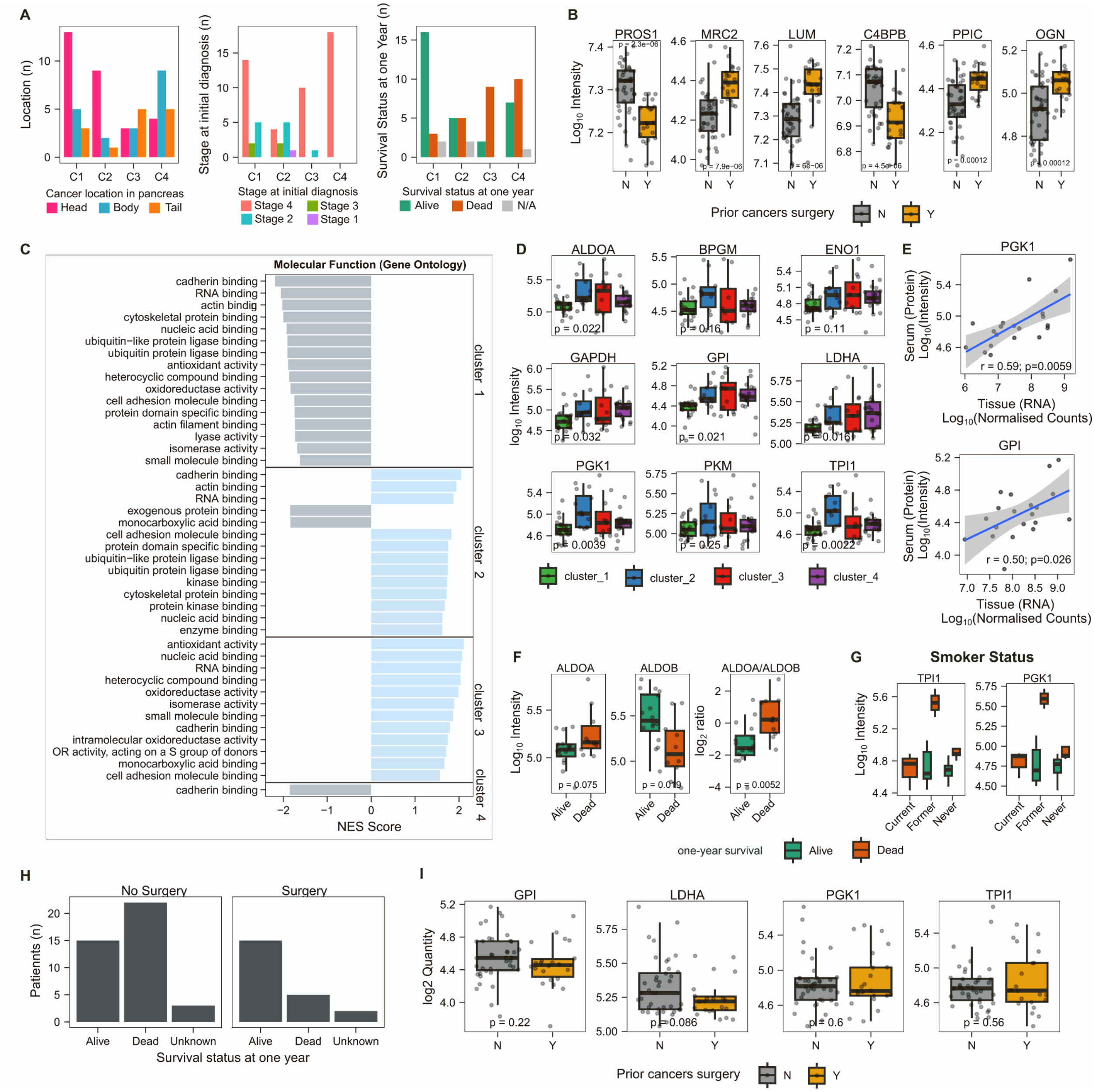

Figure S3, related to Figure 3: A) Associations of metadata with the observed clusters. Metadata includes cancer location (head, body or tail), survival at one year (Alive, Dead, or N/A), and stage at initial diagnosis (Stage 1,

Stage 2, Stage 3 or Stage 4). Number of patients in each cluster are shown as percentages (n = 62). **B)** Most significantly altered proteins between patients with and without prior surgery (two-sided t-test)**.** Boxplots show the first and third quartiles, as well as the median (thick line); whiskers extend to the most extreme data point no more than 1.5× the interquartile range from the box. **C) Changes in molecular functions (Gene Ontology) for the different clusters.** Gene Set Enrichment Analysis (GSEA) was performed for each of the clusters (Figure 2A) using molecular functions (gene ontology) as genesets. The ranked gene lists were generated by comparing the protein intensities of the samples from the respective cluster to the protein intensities of all other samples (fold-changes). Terms enriched with an adjusted p-value below 0.05 are shown. Abbreviations: OR: oxidoreductase; S gr.: sulfur group. **D) Glycolysis / Gluconeogenesis differences across patient subgroups (pretreatment protein intensities)**. Proteins that contributed most to the enrichment result (core enrichment) are shown. P-values show ANOVA results (not adjusted). The Y-axis is $\log_{10}$ transformed. The first and third quartiles, as well as the median (thick line), are shown with boxplots; whiskers extend to the most extreme data point that is no more than 1.5× the interquartile range from the box. **E) Correlation of protein levels (serum) and RNA expression (tissue biopsies).** Pearson correlation coefficient (r) and respective p-value are shown. RNA data was reanalysed from Padrón et al.[4]. Shaded area represent 95% confidence interval. **F) ALDOA / ALDOB ratio as a pretreatment biomarker for survival in Nivolumab-treated patients.** Y-axis is $\log_{10}$ transformed for ALDOA and ALDOB and $\log_2$ transformed for the ratio. P-values were calculated with a t-test (not adjusted). The first and third quartiles, as well as the median (thick line), are shown with boxplots; whiskers extend to the most extreme data point that is no more than 1.5× the interquartile range from the box. **G) Relation between smoker status, metabolism, and survival.** Pretreatment levels of TPI1 and PGK1 are shown for patients with different smoker statuses (x-axis) and different one-year survival (color). The Y-axis is $\log_{10}$ transformed. The first and third quartiles, as well as the median (thick line), are shown with boxplots; whiskers extend to the most extreme data point that is no more than 1.5× the interquartile range from the box. **H) Survival status at one year for patients without (left panel) and with (right panel) prior surgery.** Data includes both treatment arms. **I) GPI, LDHA, PGK1, and TPI are not significantly affected by prior surgery**. Log_{10} Intensities are shown for patients with and without prior surgery. Boxplots show the first and third quartiles, as well as the median (thick line); whiskers extend to the most extreme data point no more than 1.5× the interquartile range from the box.

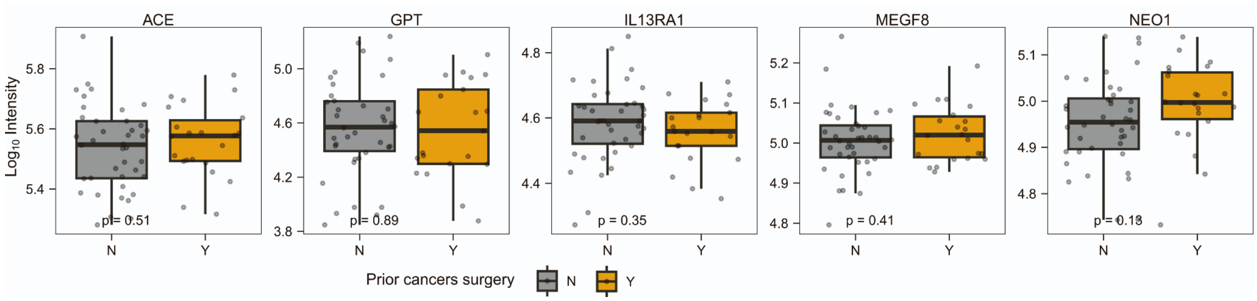

Figure S4, related to Figure 4: Proteins most relevant for predicting one-year survival in Nivolumab-treated patients are not significantly affected by prior surgery. Log_{10} Intensities are shown for patients with and without prior surgery. Boxplots show the first and third quartiles, as well as the median (thick line); whiskers extend to the most extreme data point no more than 1.5× the interquartile range from the box.

Table S1: Significant protein changes on day 15 of the first treatment cycle compared to baseline (C1D1 / C1D15).

Uniprot ID	Gene name	Adj. p-value	Log2 fold change
P54108	CRISP3	0.0000000	0.71
Q9UNW1	MINPP1	0.0000018	-0.31
Q9Y6R7	FCGBP	0.0000028	-0.67
Q14766	LTBP1	0.0000167	0.76
P48637	GSS	0.0000262	-0.72
P16930	FAH	0.0000423	-0.79
P14780	MMP9	0.0001897	1.03
P08637	FCGR3A	0.0003646	-0.66
O43852	CALU	0.0003958	0.67
P29279	CCN2	0.0004444	0.64
P13796	LCP1	0.0007406	-0.34
P40197	GP5	0.0007756	0.71
P02788	LTF	0.0008485	1.08
P12955	PEPD	0.0008637	-0.30
P02775	PPBP	0.0009499	0.57
Q9Y5Y7	LYVE1	0.0014338	-0.42
P12259	F5	0.0019525	-0.28
Q9GZT8	NIF3L1	0.0024232	-0.50
P61626	LYZ	0.0024948	0.30
P05067	APP	0.0027608	0.54
P14207	FOLR2	0.0058255	-0.32
P09486	SPARC	0.0058255	0.45

Q14112	NID2	0.0058412	0.66
P00390	GSR	0.0063241	-0.34
Q9UEW3	MARCO	0.0063241	-0.46
P49913	CAMP	0.0065475	0.83
P24298	GPT	0.0066212	-0.88
P02671	FGA	0.0086980	0.46
P05062	ALDOB	0.0090245	-0.75
P01011	SERPINA3	0.0090245	-0.29
P13727	PRG2	0.0092147	-0.61

Table S2: Proteins differentially expressed between patients receiving Nivolumab and Sotigalimab (Sotigalimab/Nivolumab). Samples were compared at day 15 of the first treatment cycle (C1D15).

Uniprot ID	Gene name	Adj. p-value	Log2 fold-change
P09486	SPARC	0.003006	-0.85372
P10619	CTSA	0.003903	-1.17423
Q14766	LTBP1	0.003903	-0.96662
P02775	PPBP	0.006376	-0.90977
O94919	ENDOD1	0.031197	-0.76961
Q13201	MMRN1	0.046128	-0.89189
P05067	APP	0.048104	-0.78211
Q9Y251	HPSE	0.063612	-0.94826
P40197	GP5	0.063612	-0.90407
Q15762	CD226	0.089209	-0.70863
P05121	SERPINE1	0.091283	-0.5816
O43852	CALU	0.091283	-0.7799

Table S3: Protein changes after 2 treatment cycles compared to baseline (C1D1 / C3D1).

Uniprot ID	Gene name	Adj. p-value	Log2 fold-change
Q9Y5Y7	LYVE1	2.86E-06	-0.57076
P13727	PRG2	9.16E-06	-0.76366
Q9NPY3	CD93	1.53E-05	-0.42353
Q9UNW1	MINPP1	1.81E-05	-0.29512
Q9Y6R7	FCGBP	4.31E-05	-0.65166
Q14314	FGL2	7.48E-05	-0.59739
Q9UBX1	CTSF	7.48E-05	-0.54721
Q03167	TGFBR3	7.48E-05	-0.44392
P08253	MMP2	0.000124	-0.33962
P35443	THBS4	0.000156	-0.47589
Q8IYS5	OSCAR	0.000311	-0.45432
Q92859	NEO1	0.000311	-0.31699
P61626	LYZ	0.000315	-0.37202
P16070	CD44	0.00091	-0.33814
P02743	APCS	0.000956	0.339188
Q9UEW3	MARCO	0.001162	-0.56512
Q9NY97	B3GNT2	0.001473	-0.22856
P07357	C8A	0.001755	0.201804
Q9UJJ9	GNPTG	0.003436	-0.20956
P02671	FGA	0.003775	0.534863
P04004	VTN	0.003997	0.13608
P25774	CTSS	0.004914	-0.36479
Q92520	FAM3C	0.00502	-0.2912
Q14624	ITIH4	0.00502	0.163668
P07360	C8G	0.005173	0.178724
Q9NZK5	ADA2	0.005306	-0.42123
Q04756	HGFAC	0.005306	-0.26191

Q8N149	LILRA2	0.006093	-0.61379
Q9Y646	CPQ	0.006773	-0.32573
Q8TER0	SNED1	0.006871	-0.25073
Q8N3T6	TMEM132C	0.006871	-0.4494
Q86YT9	JAML	0.007123	-0.42199
P19320	VCAM1	0.007123	-0.28441
P32942	ICAM3	0.007123	-0.32206
O15394	NCAM2	0.007123	-0.29455
Q8N6C8	LILRA3	0.007123	-0.62188
Q6UY14	ADAMTSL4	0.008906	-0.27037
P12318	FCGR2A	0.008906	-0.42852
Q9UM47	NOTCH3	0.009713	-0.34387
Q12907	LMAN2	0.009713	-0.28873

Table S4: 30 most changed proteins between clusters (ANOVA).

Uniprot ID	Genename	Adj. p-value
P05452	CLEC3B	3.9e-15
P06396	GSN	1.6e-12
Q9UBQ6	EXTL2	7.1e-09
P55290	CDH13	7.1e-09
Q96KN2	CNDP1	8.7e-09
P01033	TIMP1	8.7e-09
P05362	ICAM1	9.1e-09
P29622	SERPINA4	1.8e-08
Q6EMK4	VASN	2e-08
Q12860	CNTN1	5.1e-08
P11362	FGFR1	7.7e-08

P35858	IGFALS	7.7e-08
Q96PD5	PGLYRP2	1.2e-07
Q9BXJ4	C1QTNF3	1.6e-07
Q9Y279	VSIG4	3.4e-07
Q8WWZ8	OIT3	3.6e-07
P08253	MMP2	4e-07
P51884	LUM	4.4e-07
P17936	IGFBP3	4.4e-07
P27797	CALR	6.6e-07
P04003	C4BPA	9e-07
P24593	IGFBP5	1.3e-06
Q9Y624	F11R	1.3e-06
Q92743	HTRA1	1.3e-06
Q99972	MYOC	1.5e-06
P01011	SERPINA3	1.5e-06
P05154	SERPINA5	1.7e-06
Q13332	PTPRS	2.8e-06
P43121	MCAM	2.9e-06
Q9NQ79	CRTAC1	7.1e-06

Table S5: Pretreatment protein levels in Nivolumab treated patients and survival. Proteins in pretreatment samples that are significantly different between patients who survived longer than 1 year compared to patients who died within the first year.

Uniprot ID	Gene name	Adj. p-value	Log2 fold-change
O15335	CHAD	0.005465	1.763889
Q9UBG0	MRC2	0.013542	0.813022
P12821	ACE	0.016316	0.650423
P51884	LUM	0.035736	0.543044
Q92859	NEO1	0.035736	0.541457

Q12860	CNTN1	0.035736	0.672234

References

1. Uhlén, M., Fagerberg, L., Hallström, B.M., Lindskog, C., Oksvold, P., Mardinoglu, A., Sivertsson, Å., Kampf, C., Sjöstedt, E., Asplund, A., et al. (2015). Proteomics. Tissue-based map of the human proteome. Science *347*, 1260419.

2. Uhlén, M., Karlsson, M.J., Hober, A., Svensson, A.-S., Scheffel, J., Kotol, D., Zhong, W., Tebani, A., Strandberg, L., Edfors, F., et al. (2019). The human secretome. Sci. Signal. *12*. https://doi.org/10.1126/scisignal.aaz0274.

3. Rashid, N.U., Peng, X.L., Jin, C., Moffitt, R.A., Volmar, K.E., Belt, B.A., Panni, R.Z., Nywening, T.M., Herrera, S.G., Moore, K.J., et al. (2020). Purity Independent Subtyping of Tumors (PurIST), A clinically robust, single-sample classifier for tumor subtyping in pancreatic cancer. Clin. Cancer Res. *26*, 82–92.

4. Padrón, L.J., Maurer, D.M., O'Hara, M.H., O'Reilly, E.M., Wolff, R.A., Wainberg, Z.A., Ko, A.H., Fisher, G., Rahma, O., Lyman, J.P., et al. (2022). Sotigalimab and/or nivolumab with chemotherapy in first-line metastatic pancreatic cancer: clinical and immunologic analyses from the randomized phase 2 PRINCE trial. Nat. Med. *28*, 1167–1177.
